# Supplementary material for: Obesity and smoking as risk factors for invasive mechanical ventilation in COVID-19: A retrospective, observational cohort study
Source: PLoS One. 2020 Dec 22;15(12):e0238552. doi: 10.1371/journal.pone.0238552 (PMC7755188; doi:10.1371/journal.pone.0238552)
Supplement: S1 File — (PDF) [file pone.0238552.s001.pdf]

Supplemental Table 1. Experimental Therapies Administered to Cohort Patients	
**Medication**	**Number of patients**
No Medication	52
**Individual Therapies**	
Anti-IL-6	3
Remdesivir	6
Leronlimab	6
Hydroxychloroquine	15
**Combination Therapies**	
Anti-IL-6 and Remdesivir	1
Anti-IL-6 and Leronlimab	3
Anti-IL-6 and Hydroxychloroquine	14
Remdesivir and Leronlimab	1
Remdesivir and Hydroxychloroquine	2
Leronlimab and Hydroxychloroquine	2
Anti-IL-6 and Leronlimab and Hydroxychloroquine	1
Anti-IL-6 and Remdesivir and Hydroxychloroquine	5
Anti-IL-6 and Remdesivir and Leronlimab and Hydroxychloroquine	1
**Anti-IL 6 therapies include tocilizumab and sarilumab. Patients in these groups received only one type.*	

Supplemental Table 2: Interventions administered open label or via trial enrollment. Those enrolled in clinical trial received either the listed medication or placebo in a blinded fashion.			
**Medication**	**Total**	**% in Trial**	**% Open Label**
**Hydroxychloroquine**	40	0%	100%
**Leronlimab**	14	7%	93%
**Remdesivir (or placebo)**	16	88%	22%
**Sarilumab (or placebo)**	9	100%	0%
**Tocilizumab**	19	21%	79%

Supplemental Table 3: Reported Indication for Therapeutic Dose Anticoagulation	
**Anticoagulation Indication**	**N**
Acute Coronary Syndrome	1
Atrial Fibrillation	6
ECMO	1
Empiric for <24 hours	1
Frequent dialysis line clotting	2
Ischemic toe	1
LV Thrombus	1
New in-hospital VTE	6
Past Medical History (includes prior VTE)	7

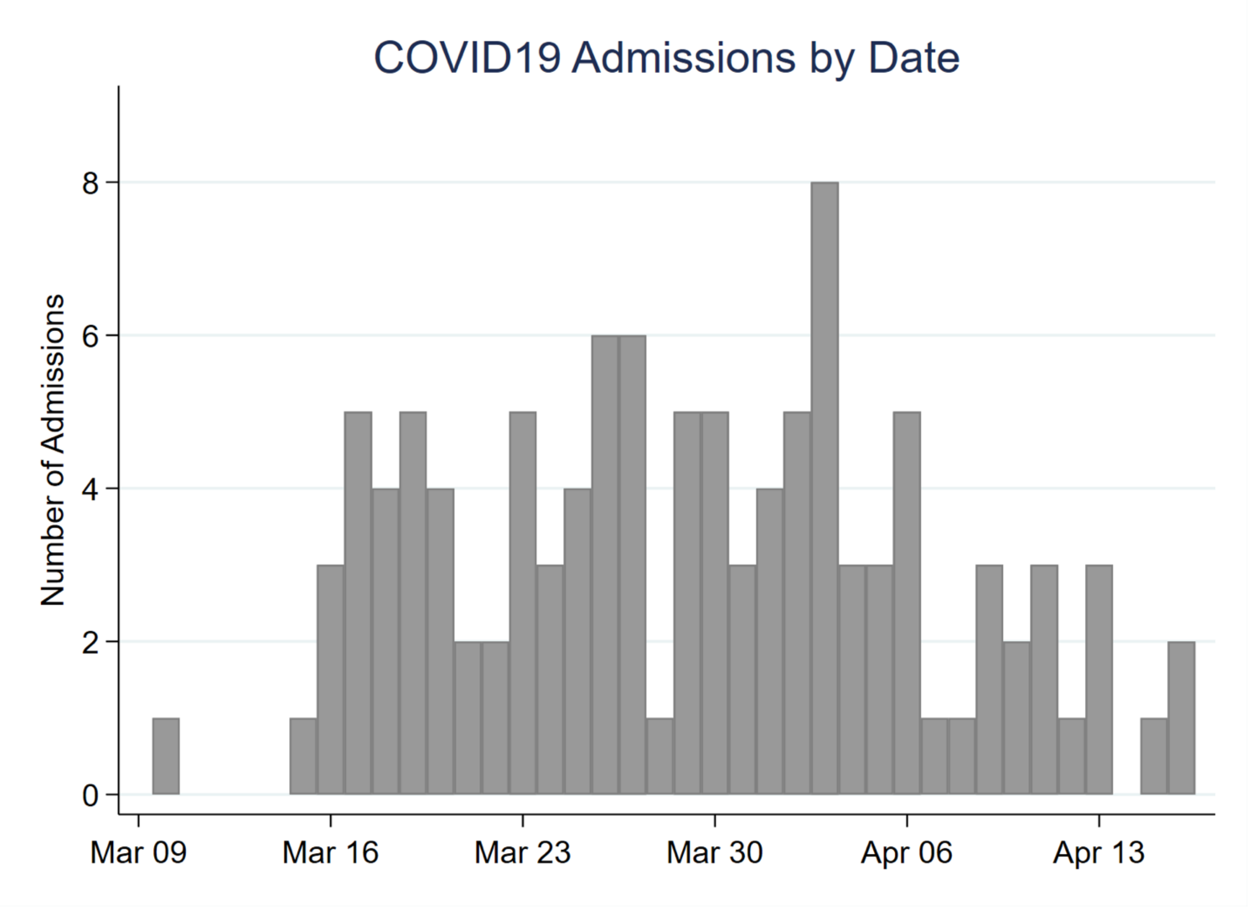

Supplemental Figure 1: Combined COVID-19 admissions by date for both Ronald Reagan UCLA and Santa Monica UCLA. Of note, one admission from December and one from February, both of whom contracted COVID-19 during hospitalization were omitted from this histogram for ease of interpretation.
