# Supplementary material for: Obesity and smoking as risk factors for invasive mechanical ventilation in COVID-19: A retrospective, observational cohort study
Source: PLoS One. 2020 Dec 22;15(12):e0238552. doi: 10.1371/journal.pone.0238552 (PMC7755188; doi:10.1371/journal.pone.0238552)
Supplement: Attachment — Submitted filename: Response to Reviewers.docx [file pone.0238552.s002.docx]

**Response to Reviewers**

1. Please ensure that your manuscript meets PLOS ONE's style requirements, including those for file naming. The PLOS ONE style templates can be found at

[https://journals.plos.org/plosone/s/file?id=wjVg/PLOSOne_formatting_sample_main_body.pdf](https://urldefense.com/v3/__https:/journals.plos.org/plosone/s/file?id=wjVg*PLOSOne_formatting_sample_main_body.pdf__;Lw!!F9wkZZsI-LA!UE1Ks4kghsvLcsFits1ugFq2Vxa3JD_VqK97R9DiuYmYir1ZkcqdHXRXaJlIDiDeiUUjzqsJJQ$) and

[https://journals.plos.org/plosone/s/file?id=ba62/PLOSOne_formatting_sample_title_authors_affiliations.pdf](https://urldefense.com/v3/__https:/journals.plos.org/plosone/s/file?id=ba62*PLOSOne_formatting_sample_title_authors_affiliations.pdf__;Lw!!F9wkZZsI-LA!UE1Ks4kghsvLcsFits1ugFq2Vxa3JD_VqK97R9DiuYmYir1ZkcqdHXRXaJlIDiDeiUU-F55Fbw$)

*We have revised the manuscript to comply to the above guidelines.*

**2. Please include the date(s) on which you accessed the databases or records to obtain the data used in your study.**

*We have added the following sentence to the manuscript methods section:*

*“This is a retrospective observational cohort study conducted from March 12 2020 and April 16 2020 approved by the UCLA (University of California, Los Angeles) institutional review board with waiver of informed consent. We evaluated the first 113 unique admissions to our healthcare system. We originally accessed patient charts between March 12 and June 16^th^ 2020. We returned to the charts in October 2020 to extract elements requested during the review process.”*

**3. Please define current, former, and never smoker statuses.**

*We have added these definitions to the methods section:*

*“We extracted smoking status from the admission note. Patients were interviewed about smoking history on admission. Never smokers, prior smokers and current smokers were self-identified as such by the patients during the interview. Those patients who self-identified as prior smokers were then asked when they last smoked and number of packs a day. Those who identified as current smokers were asked the number of packs smoked a day. Patients who smoked less than a pack-year were considered never smokers.”*

**4. Thank you for stating the following in the Competing Interests section: "I have read the journal's policy and the authors of this manuscript have the following competing interests: SYC consults for PureTech on their deupirfenidone in COVID study. "**

**Please confirm that this does not alter your adherence to all PLOS ONE policies on sharing data and materials, by including the following statement: "This does not alter our adherence to PLOS ONE policies on sharing data and materials.” (as detailed online in our guide for authors** [**http://journals.plos.org/plosone/s/competing-interests**](about:blank)**).  If there are restrictions on sharing of data and/or materials, please state these. Please note that we cannot proceed with consideration of your article until this information has been declared.**

**Please include your updated Competing Interests statement in your cover letter; we will change the online submission form on your behalf.**

**Please know it is PLOS ONE policy for corresponding authors to declare, on behalf of all authors, all potential competing interests for the purposes of transparency. PLOS defines a competing interest as anything that interferes with, or could reasonably be perceived as interfering with, the full and objective presentation, peer review, editorial decision-making, or publication of research or non-research articles submitted to one of the journals. Competing interests can be financial or non-financial, professional, or personal. Competing interests can arise in relationship to an organization or another person. Please follow this link to our website for more details on competing interests:** [**http://journals.plos.org/plosone/s/competing-interests**](about:blank)

*We have added the following to our cover letter:*

*“I have read the journal's policy and the authors of this manuscript have the following competing interests: SYC consults for PureTech on their deupirfenidone in COVID study. This does not alter our adherence to PLOS ONE policies on sharing data and materials.”*

**5. We note that you have indicated that data from this study are available upon request. PLOS only allows data to be available upon request if there are legal or ethical restrictions on sharing data publicly. For information on unacceptable data access restrictions, please see** [**http://journals.plos.org/plosone/s/data-availability#loc-unacceptable-data-access-restrictions**](https://urldefense.com/v3/__http:/journals.plos.org/plosone/s/data-availability*loc-unacceptable-data-access-restrictions__;Iw!!F9wkZZsI-LA!UE1Ks4kghsvLcsFits1ugFq2Vxa3JD_VqK97R9DiuYmYir1ZkcqdHXRXaJlIDiDeiUVzytVtkA$)**.**

**In your revised cover letter, please address the following prompts:**

**a) If there are ethical or legal restrictions on sharing a de-identified data set, please explain them in detail (e.g., data contain potentially identifying or sensitive patient information) and who has imposed them (e.g., an ethics committee). Please also provide contact information for a data access committee, ethics committee, or other institutional body to which data requests may be sent.**

**b) If there are no restrictions, please upload the minimal anonymized data set necessary to replicate your study findings as either Supporting Information files or to a stable, public repository and provide us with the relevant URLs, DOIs, or accession numbers. Please see** [**http://www.bmj.com/content/340/bmj.c181.long**](https://urldefense.com/v3/__http:/www.bmj.com/content/340/bmj.c181.long__;!!F9wkZZsI-LA!UE1Ks4kghsvLcsFits1ugFq2Vxa3JD_VqK97R9DiuYmYir1ZkcqdHXRXaJlIDiDeiUWosH8ltA$) **for guidelines on how to de-identify and prepare clinical data for publication. For a list of acceptable repositories, please see** [**http://journals.plos.org/plosone/s/data-availability#loc-recommended-repositories**](https://urldefense.com/v3/__http:/journals.plos.org/plosone/s/data-availability*loc-recommended-repositories__;Iw!!F9wkZZsI-LA!UE1Ks4kghsvLcsFits1ugFq2Vxa3JD_VqK97R9DiuYmYir1ZkcqdHXRXaJlIDiDeiUWXbuwPZA$)**.**

*We have since uploaded the data used in the manuscript in the public repository* [*https://datadryad.org/stash*](https://datadryad.org/stash)*. The DOI is* https://doi.org/10.5068/D1QX18

**We will update your Data Availability statement on your behalf to reflect the information you provide.**

**6. While revising your submission, please upload your figure files to the Preflight Analysis and Conversion Engine (PACE) digital diagnostic tool,** [**https://pacev2.apexcovantage.com/**](https://urldefense.com/v3/__https:/pacev2.apexcovantage.com/__;!!F9wkZZsI-LA!UE1Ks4kghsvLcsFits1ugFq2Vxa3JD_VqK97R9DiuYmYir1ZkcqdHXRXaJlIDiDeiUWOfS6_ZQ$)**. PACE helps ensure that figures meet PLOS requirements. To use PACE, you must first register as a user. Registration is free. Then, login and navigate to the UPLOAD tab, where you will find detailed instructions on how to use the tool. If you encounter any issues or have any questions when using PACE, please email PLOS at** [**figures@plos.org**](mailto:figures@plos.org)**. Please note that Supporting Information files do not need this step.**

*Thank you, we have completed this.*

**In Response to reviewers' comments:**

**Reviewer #1: The goal of this study was to evaluate respiratory failure in COVID-19 and explore factors associated with risk of invasive mechanical ventilation (IMV). A retrospective, observational cohort study of 112 inpatient adults diagnosed with COVID-19 was carried out. Data were manually extracted from electronic medical records. Multivariable and Univariable regression were used to evaluate association between baseline characteristics, initial serum markers and the outcome of IMV. It was determined that obesity, smoking history, and elevated inflammatory markers were associated with increased need for IMV in patients with COVID-19. These findings are of utmost importance given the current scenario.**

*We appreciate your support of our manuscript.*

**Reviewer #2: In this study (PONE-D-20-26498), Monteiro and colleagues described a small sample (28), who needed endotracheal intubation and invasive mechanical ventilation, among 112 COVID-19 included in the analysis. The idea is interesting the paper is well written, however I have some considerations to explain. First of all they described that 28 patients out 47 admitted to the ICU were intubated, but they did not describe the criteria of intubation.
Could you describe the intubation criteria that you used, please?**

*Thank you for pointing this out. In the methods we have now added the following statement:*

*“Guidelines recommended intubation for patients who had PCR confirmed COVID-19 and who demonstrated rapid escalation of oxygen requirements. It was at the discretion of the clinician to decide whether a patient would tolerate a trial of non-invasive oxygen delivery via non-rebreather mask or high flow nasal canula (HFNC). If the clinician decided that trial of these non-invasive therapies would place the patient and/or staff at greater risk, the patient would proceed to intubation.”*

**Moreover they did not describe any severity score, i.e. APACHE II, SAPS II. Could you add a severity score to understand better the severity of your population admitted to ICU, since your mortality rate was very low compared to other ICU?**

*We appreciate the suggestion. We re-evaluated the COVID literature and found a recent study on COVID-19 patients from Wuhan which reported that APACHE II outperformed SOFA and CURB-65 as a predictor of mortality in this population (*[*https://www.ncbi.nlm.nih.gov/pmc/articles/PMC7217128/*](https://www.ncbi.nlm.nih.gov/pmc/articles/PMC7217128/)*). As such, we chose to calculate APACHE II for the patients in our cohort who were admitted to the ICU. We have added the following to the results sections:*

*“Out of the 47 ICU admissions, the median APACHE II score on arrival to the ICU was 12 (IQR 7-16, mean 12.7, SD 7.11).”*

*In comparison, the cohort from Wuhan, China had a mean APACHE score of 15 +- SD 7.7 (*[*https://www.ncbi.nlm.nih.gov/pmc/articles/PMC7217128/*](https://www.ncbi.nlm.nih.gov/pmc/articles/PMC7217128/)*). Considering the population size, standard deviation and means of these two populations, a two tailed t-test revealed that the APACHE scores in these two populations were not significantly different.*

**Regarding mechanical ventilation, they described a setting of median PEEP level of 10-12 cmH2O in severe ARDS and P/F ratio < 150, but they did not describe any data on plateau pressure, driving pressure or compliance of the respiratory system. Therefore how did you decide to set PEEP level? Could you describe better respiratory mechanics (plateau pressure, driving pressure, PEEP, compliance of the respiratory system)? Which criteria did you consider to decide for prone positioning? Could you report the duration of mechanical ventilation?**

*As stated in our methods, we recommended PEEP titration per ARDS network PEEP tables and prone positioning when P/F <150, as recommended by the PROSEVA trial: “…early consideration of prone ventilation for patients with P/F ratios < 150” and “Titration of positive end-expiratory pressure (PEEP) was recommended to reflect ARDS network PEEP tables”.*

*We have also added that we recommended PPlat to stay under 30 cm H_2_O.*

*We have since included the static compliance of patients who were intubated, as measured as part of our respiratory therapy routine care. We have added the following to our manuscript:*

*“ For the intubated patients, compliance on day of intubation (day 1) had a median value of 34.1 ml/cm H_2_O (IQR = 24.6-41.8, mean 34.0, SD 10.4), for day 3 the median was 30.3 (IQR 23.9-40.1, mean 32.7, SD 10.4), for day 5 the median was 34.6 (IQR 26.9-39.9, mean 35.4, SD 12.4) and for day 7 the median was 34.7 (IQR 30.7-52.5, mean 38.8, SD 11.7).”*

*Interestingly, the static compliance measured in these patients were most similar to the non COVID ARDS arm reported in the Grasselli study (https://www.thelancet.com/journals/lanres/article/PIIS2213-2600%2820%2930370-2/fulltext), in which they reported a median compliance of 32 ml/cm H2O (IQR 25-43), and lower than the median of 41 reported for the COVID-19 arm of the same study.*
